# Ethnic Bias after Ethnic Conflict: Preferential Voting and the Serb Minority in Croatian Elections

**DOI:** 10.1080/17449057.2021.1997440

**Published:** 2021-11-17

**Authors:** Josip Glaurdić, Michal Mochtak, Christophe Lesschaeve

**Affiliations:** Institute of Political Science, University of Luxembourg, Esch-sur-Alzette, Luxembourg

## Abstract

In spite of growing interest in democratization and electoral competition after ethnic conflict, we know little about the impact of ethnic violence on voter choice in post-conflict societies. This article uses an original dataset of local-level electoral results, communities’ exposure to war violence, and candidates’ ethnicity derived from names in contemporary Croatia to uncover the relationship between local post-conflict ethnic distribution, ethnic violence, and the electorate's ethnic bias. Our analysis points to the presence of ethnic bias that is determined by local interethnic balance and exposure to war violence – particularly for communities populated by the Serb minority.

A long line of research shows that voters value candidates who match their sociodemographic characteristics – whether it is gender, race, religion, or ethnicity (Heath et al., 2015; Philpot & Walton, 2007; Van Erkel, 2019). In many ways, this preference for representation of social identity is understandable. We tend to believe, rightfully or not, that candidates who look like us are more likely to have the same policy preferences as we do and to represent more faithfully our interests, as well as the interests of our social groups. In other words, we tend to believe that descriptive representation will almost inherently lead to substantive representation (Phillips, 1995; Pitkin, 1967). This kind of voter decision-making, however, can lead to serious underrepresentation of minorities – depending on the electoral rules in place and the geographic distribution of ethnic groups. The problem becomes even greater in societies with heightened levels of politicized identity and recent history of identity-based conflict. In this article, we focus on the subject of ethnic bias in polities still recovering from the consequences of ethnically based violent conflict. Our analysis addresses several closely related questions. Are voters in societies recovering from ethnic conflict ethnically biased? Is this bias more prevalent among the members of the ethnic majority or minority? Is it uniformly distributed or does it depend on the local balance between the ethnic groups? Finally, what is the impact of the electorate’s exposure to ethnically based conflict violence on its post-conflict ethnic bias? Are communities that were more exposed to inter-ethnic violence more ethnically biased after the conflict has ended?

We answer these questions using a unique dataset combining characteristics of more than five thousand electoral candidates, results of three rounds of elections conducted under proportional representation rules with preferential voting in the period between 2015 and 2020, and a string of sociodemographic and war violence data on the level of more than five hundred municipalities in contemporary Croatia. Our attention is focused on the electoral fortunes of Serb candidates due to the recent history of violent conflict between the Serb minority and the Croat majority in the Croatian 1991–1995 War for Independence. What sets our analysis apart from similar studies of voter ethnic bias is the post-conflict context, the granularity and quality of our data, as well as a novel approach we developed for inferring candidates’ ethnicity from their names using a machine-learning algorithm trained on a sample of more than 225,000 people from all Croatian regions.

The results of our analysis point to the presence of ethnic bias that is determined by the local environment. Serb candidates are slightly penalized in areas with Croat majorities and considerably rewarded in areas with Serb majorities, though we observe a decreasing rate of return to candidates’ Serb ethnicity as the proportion of Serbs in the electorate increases – a sign that ethnic bias is likely more prevalent among minority voters in areas of ethnic polarization. We use pre-war survey evidence on ethnic prejudice among Croats and Serbs in different regions of Croatia and conclude that the possibility of reverse causality (i.e. pre-war ethnic bias causing war violence) is unlikely. We also find the relationship between the ethnic identity of the local electorate and its ethnic bias to be conditional on the level of exposure to war violence. Our analysis shows that for Serb communities, war exposure dramatically increases their electoral ethnic bias. The same, however, cannot be said for Croat communities. We believe this is the case because ethnically based violence intensifies the process of withdrawal into a community of co-ethnics among ethnic minority voters who have more reason to feel insecure in the post-conflict environment of ethnic tension and majority’s efforts at retribution through administrative and other discrimination. Considering the fact that we examine elections taking place more than two decades after the war, our findings are also a testament to the depth and long-lasting nature of consequences ethnic conflict can have on interethnic relations and the challenge of rebuilding the bonds of interethnic trust needed for a functioning democracy.

## Ethnic Bias in Voter Choice and Post-Conflict Ethnicity

Explanations of ethnic bias in voting usually come in two forms: one that sees this bias in voter choice as an expression of identity and one that perceives it to be a consequence of the voters’ rational, instrumental calculation about the candidates’ policy preferences. Proponents of the expressive/identity school of thought see voting as an opportunity for individuals to signal their affiliation with and allegiance to a social and cultural group (Ferree, 2006; Green et al., 2002; Huddy et al., 2015). Voters, thus, may show ethnic bias in their choice because of a common sense of belonging with (or because of ethnic animus or prejudice against) the candidates. Proponents of the rational/instrumental school of thought, on the other hand, see candidates’ ethnicity as an information shortcut to their policy views (Johnston et al., 1992; Popkin, 1991). Different ethnic groups may have different policy preferences, particularly in societies with histories of ethnically based competition and overlapping socioeconomic cleavages. Candidates’ ethnicity, therefore, could be seen as a straightforward cue for their propensity to represent the ethnic group’s interests (McConnaughy et al., 2010).

Whatever the underlying reasons may be, ethnic bias in voting is a phenomenon that has been observed in a multitude of geographic, temporal, and institutional contexts. In the United States, for example, both white and minority voters have been shown to make ethnically/racially biased voting decisions, with white voters’ choices in part driven by their attitudes toward candidates’ different ethnic/racial backgrounds (Abrajano & Hajnal, 2015; Tesler & Sears, 2010) and the minority voters being drawn to candidates matching their race/ethnicity (Boudreau et al., 2019). Results are similar in the UK, where minority candidates of non-European origin are penalized at the ballot box, especially by white voters with anti-immigrant sentiments (Fisher et al., 2014), and minority candidates of European origin are penalized in districts disproportionately populated by minority voters of non-European origin (Thrasher et al., 2015). The evidence of ethnically biased voting seems to be particularly strong among ethnic minority voters. Minority voters have been found to be biased in favor of co-ethnics in electoral competitions in Belgium (Teney et al., 2010), Canada (Landa et al., 1995), Denmark and Norway (Bergh & Bjørklund, 2003), India (Heath et al., 2015), and the United States (Boudreau et al., 2019). These findings fit well within the larger literature on descriptive or social representation that has shown voters to prefer candidates who match their own gender (Van Erkel, 2019), class (Heath, 2015), or race (Philpot & Walton, 2007).

There is reason to believe, however, that this relationship between ethnic identity and ethnic bias is not necessarily linear, but is in fact dependent on local contextual factors such as candidates’ winning prospects, electoral rules, or local interethnic balance (Heath et al., 2015; Ichino & Nathan, 2013). In our view, this line of evidence resonates particularly well with the insights from the literature on ethnic polarization and ethnic conflict (Montalvo & Reynal-Querol, 2005). Ethnic polarization, i.e. the context in which two ethnic groups are closely numerically balanced, has been found to be a good predictor of the onset, intensity, and length of conflict in studies with both countries and sub-national local communities as cases (Costalli & Moro, 2012; Esteban et al., 2012; Montalvo & Reynal-Querol, 2010). Translated into the electoral arena, these findings suggest it would be reasonable to expect voters in ethnically polarized areas having a greater propensity to support co-ethnic candidates due to the local environment of interethnic competition for control over limited public goods.

The critical question of our interest, however, concerns the possible impact of exposure to war violence on ethnic bias. Some survey-based research focused on the Balkans suggests that ethnic animus in the region dissipated rather quickly after the war (e.g. Dyrstad, 2012; Strabac & Ringdal, 2008). The body of evidence suggesting the opposite, however, is far more convincing. As Elisabeth Wood (2008) persuasively concludes after summarizing a growing literature on the social impact of civil conflict, violence reconfigures social networks by polarizing identities and strengthening ethnicized lines of division. Furthermore, experience of combat has been shown to radicalize and harden attitudes, making interethnic compromise less likely (Grossman et al., 2015). Exposure to violence has also been shown to decrease trust and interethnic social capital while increasing ethnic identity (Besley & Reynal-Querol, 2014; De Luca & Verpoorten, 2015). Exposure to violence has also been shown to increase support for ethnically based parties (Hadzic et al., 2020) – perhaps unsurprisingly considering the fact that elections are arenas of competition where visible identities are often (ab)used through appeals to recent violence in order to increase electoral mobilization (Wilkinson, 2004).

What is particularly important to note here is that experiences of war violence should be considered as collective, rather than individual, phenomena. A growing body of work shows that experiences of war violence become constitutive elements of collective memories (Zubrzycki & Woźny, 2020) that continue to exert their sociopolitical influence on communities – often regardless of individuals’ personal experiences of conflict. New evidence stemming from research conducted on the level of communities shows the lasting power of collective experiences of violence on post-conflict politics. Researchers focused on Southeast Europe have demonstrated the impact of exposure to war violence on electoral results in Bosnia and Herzegovina, Croatia, and Serbia: ethnically based parties in contemporary Bosnia and Herzegovina do better in areas more affected by war violence in the 1992–1995 war (Hadzic et al., 2020); the nationalist right in Croatia does better in areas more exposed to violence in Croatia’s War for Independence (Glaurdić & Vuković, 2016); and the NATO bombing of Serbia seems to have had a significant effect on the electoral results of the Milošević regime (Popović, 2021). Similarly, the geographic pattern of support for the political left in post-World War II Italy was decisively determined by the pattern of communist armed activity during the war (Costalli & Ruggeri, 2019). And the areas hit by Stalinist persecution in 1940s Ukraine are less likely to vote for pro-Russian parties even today (Rozenas et al., 2017). Despite these recent highly suggestive pieces of evidence on the long-lasting effects of exposure to violence on post-conflict politics^1^, however, we still do not understand the relationship between communities’ exposure to violence, post-conflict ethnic distribution, and voters’ ethnic bias, because we simply lack studies with sufficient quality of real-world electoral data in post-conflict environments.

**Figure 1.**
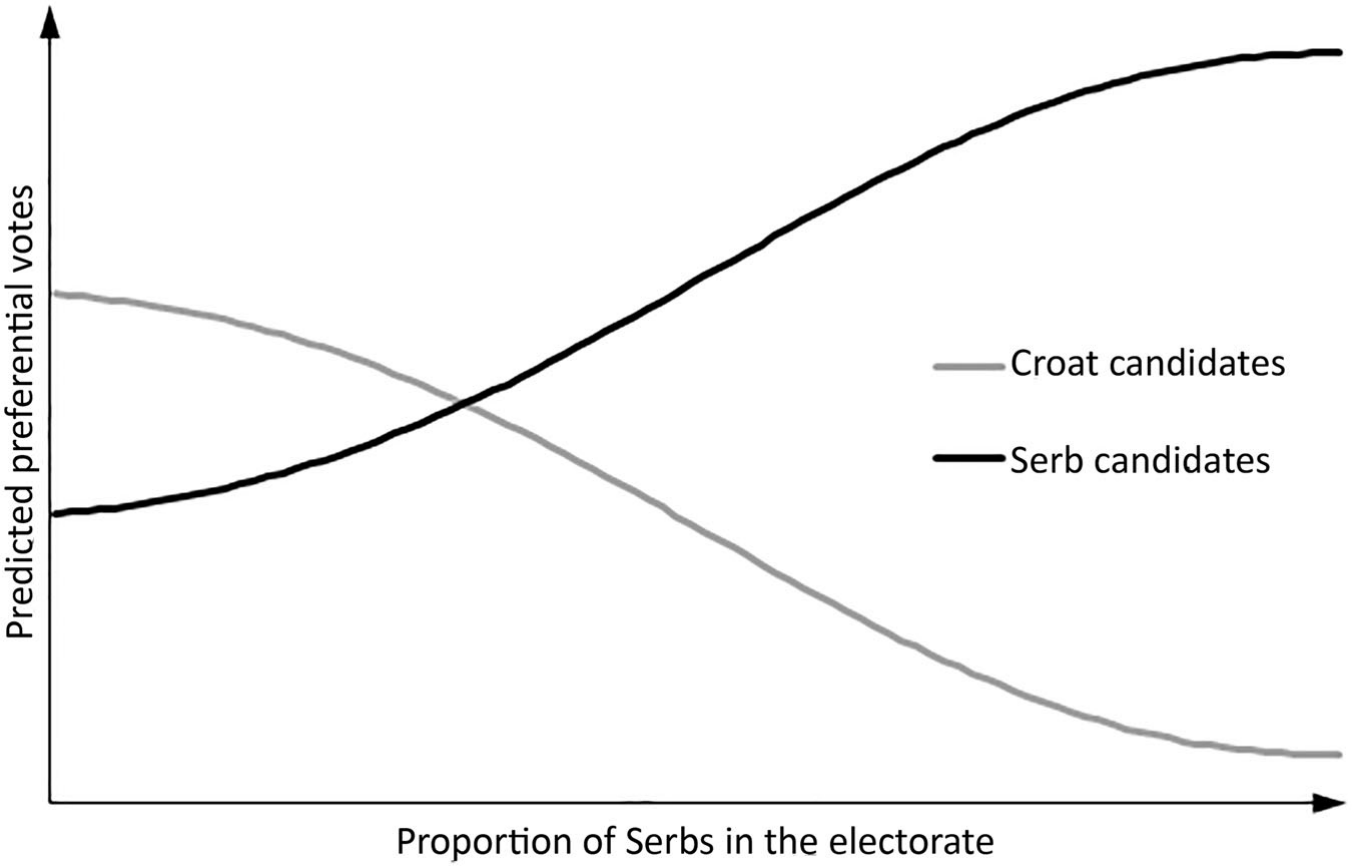
Graphical representation of the second set of empirical propositions.

Considering the general thrust of the literature on ethnic bias, as well as the literature on the effects of exposure to war violence on ethnic identity and interethnic relations, we make the following empirical propositions. First, we propose that candidates belonging to an ethnic minority (in our case, Serb candidates) will experience a lower level of electoral support in the form of preferential votes (H1). Croatia’s War for Independence may have ended two and a half decades ago, but we believe it likely that Serb candidates are penalized by the electorate that is in general overwhelmingly Croat. Second, however, we believe the electorate’s ethnic bias to be dependent on its local ethnic makeup. In simple terms, we believe minority candidates should be electorally rewarded in areas more populated by that minority and penalized in areas populated by the majority (H2) – though with two important stipulations. The first stipulation concerns the size of this electoral reward/penalty. Following the findings of the literature on ethnic bias that suggest this bias to be more pronounced among voters belonging to ethnic minorities, we believe the same should be observed in the pattern of electoral results in Croatia, i.e. the electoral bonus for Serb candidates in Serb areas should be greater than the electoral penalty they experience in Croat areas (H2a). The second stipulation concerns the shape of the reward/penalty curve: we believe it not to be linear. Following the long line of research on the relationship between ethnic distribution and interethnic conflict, we believe that ethnicity should be more salient in areas with high polarization between the ethnic majority and the principal ethnic minority. Coupled with the expectation of ethnic bias being greater among minority voters, this proposition in practical terms means we should observe something akin to the decreasing returns to candidates’ Serb ethnicity as the proportion of ethnic Serbs in the local electorate increases (H2b). For easier understanding of this set of empirical propositions, we present our expectations in graphic form in Figure 1, where the lines for predicted preferential votes for Croat and Serb candidates have been derived using simple Gaussian functions for ethnic bias for the two ethnic groups with their centers at the point of highest ethnic polarization.

Taking into account the emerging evidence of the long-lasting effects of communal exposure to war violence on ethnic identities and political affiliations, we furthermore believe our set of propositions on the relationship between the ethnicity of the local electorate and ethnic bias (i.e. H2, H2a, and H2b) should be contingent on the local level of exposure to war violence. Put more precisely, we believe the local level of exposure to war violence should intensify the level of ethnic bias in voting, and particularly in communities populated by the Serb minority. We believe this to be the case because we agree with those who argue that ethnically based violence furthers the process of seeking a safe haven within the community of co-ethnics (Hadzic et al., 2020). This process tends to outlast the actual violence and is often used as a communal coping mechanism – particularly among members of the ethnic minority who have more reason to feel insecure in the post-conflict environment of ethnic tension and likely discrimination at the hands of the ethnic majority.

If we directly reference our propositions about the relationship between the local electorate’s ethnicity and voters’ ethnic bias, ethnic minority candidates should be even more electorally rewarded in areas populated by that ethnic minority that were disproportionally exposed to war violence; they should also be even more penalized in areas populated by the ethnic majority that were disproportionally exposed to war violence (H3). The difference between the electoral bonus for minority candidates in minority areas and the electoral penalty they experience in majority areas should be greater in areas disproportionally exposed to war violence than in areas that avoided war operations (H3a). When it comes to the curve capturing the decreasing returns to candidates’ ethnic minority identity as the proportion of that ethnic minority in the local electorate increases, we believe it likely that it is flatter in areas of low war exposure and steeper in areas of high war exposure (H3b). In reference to Figure 1, we believe the two lines representing the preferential votes of minority and majority candidates should be squeezed closer together in areas of low war exposure, and more spread apart and divergent in areas of high war exposure.

## Serb Ethnic Minority in Contemporary Croatian Politics

The Croatian War for Independence (1991–1995) was the first war on European soil after the end of World War II. Battle lines were drawn between the forces of the Croatian government seeking independence from Yugoslavia on the one side and the Croatian Serbs backed by Serbia and the Yugoslav Army on the other. The rebellion of the Croatian Serbs mostly took place in areas where they constituted at least a sizeable minority. These areas were militarily occupied and declared an independent Republic of Serb Krajina (RSK). As many as a half of Croatian Serbs – living mostly in big cities – stayed loyal to the Croatian government, whether out of necessity or conviction. Many of them, however, were subjected to discrimination in their workplaces, schools, public institutions, or among friends. The war had disastrous consequences for the whole country, but particularly for the Serb community. About twenty thousand people lost their lives – some thirteen thousand on the Croatian side and seven thousand on the side of the rebel Serbs. Moreover, nearly 20% of the country’s population experienced forcible displacement, with 150,000–200,000 Croatian Serbs (about a third of all Serbs in Croatia) fleeing from the defeated RSK after Croatia’s Operation Storm effectively ended the war in August 1995.

The postwar period brought a slow and at best uneven improvement in interethnic relations. The return of Serb refugees was impeded by a number of discriminatory administrative measures of the Croatian government (Djuric, 2010). Reconciliation was not assisted by the highly politicized process of transitional justice, failures of the International Criminal Tribunal for the Former Yugoslavia (ICTY), and the resistance of political actors in the region to accept culpability. Members of the Serb minority in Croatia continue to experience discrimination to this day (Pučki pravobranitelj, 2020). Particularly notable in this respect is the public discourse of rightwing politicians who seem to have zeroed in on parliamentarians who are either Serbs or whose Croat ethnic identity is considered suspect due to their ethnically ambiguous first and last names. The annual reports of the Serb National Council – the largest cultural organization of the Croatian Serbs – methodically recounts these examples of ethnically based hate speech which seems to increase in prominence during electoral campaigns (e.g. Ponoš, 2021). Although the number of Croatian Serbs has been more than halved by the war – in 1991, they constituted about 12% of Croatia’s population and today they constitute about 5% – they still form the majority in parts of the territory that was claimed by the RSK. When it comes to their parliamentary representation, Croatian Serb voters since the first postwar elections in 1995 have been given a choice of either declaring themselves as ethnic Serbs at the polling place and then voting for three constitutionally guaranteed Serb ethnic minority representatives or voting for the parties and candidates in the regular elections as the voters of the Croat ethnic majority do.^2^

After the first three rounds of democratic elections were held under majoritarian and mixed electoral rules in the 1990s, Croatia instituted a system of proportional representation in advance of the 2000 elections. Since then, Croatian voters have been voting for party lists in ten equally sized multi-member districts with the district magnitude of 14. The proportion of Croatian Serb adults opting to vote for the three constitutionally mandated ethnic minority representatives has steadily declined from about 30% to less than 10% in the 2020 elections. In other words, an overwhelming majority of Croatian Serbs, for a variety of reasons, today chooses to vote in regular elections together with the rest of the electorate. Many Croatian Serbs also run on regular lists of Croatian parties. In advance of the 2015 election, party lists were opened and voters could subsequently allocate their preferential votes to individual candidates (Odbor za zakonodavstvo, 2015). Our study focuses on these regular elections held under PR rules with preferential voting in ten MMDs in 2015, 2016, and 2020. Elections for constitutionally mandated ethnic minority representatives are beyond our scope because they are conducted under different electoral rules. They can also shed no light on possible ethnic bias of the electorate since voters and candidates are all of the same ethnicity.

## Data and Method

More than 60% of Croatian voters cast a preference vote in the three elections relevant for our study (66.6% in 2015, 66.0% in 2016, and 62.7% in 2020) – a comparatively higher figure than in other European countries with similar electoral rules.^3^ Since the Croatian Electoral Commission publishes all results on the level of individual precincts, we were able to match vote tallies with Croatia’s relatively small 556 municipalities, which is important because of availability of comprehensive economic, sociodemographic, and war-related data on the municipal level. Although the three elections under scrutiny took place within a limited time span, candidate turnover was substantial, resulting in a sample of 5437 unique and 6669 total candidates. Our interest centers on explaining why voters choose a particular candidate over other candidates of his/her party, as well as over a list vote. This is why the dependent variable in our analyses is the vote cast for a candidate expressed as a proportion of all votes cast for the candidate’s party in a given municipality (i.e. list votes plus all preferential votes). Here we follow the standard of the literature on voter preferences in open and flexible list systems (e.g. Allik, 2015; Dettman et al., 2017; Van Erkel & Thijssen, 2016). We should note that observations in our dataset are weighted by the absolute number of votes that are behind the proportion, because it is important to ensure that case results are not skewed toward the vote dynamics observed for small parties and small municipalities.

This process of disaggregation of electoral results to the municipal level creates a stacked dataset where candidate*municipality is the unit of analysis. Since this stacking procedure expands the number of observations at each level, failing to account for it would lead to an underestimation of the regression coefficients’ standard errors, as well as an overestimation of statistical significance (Rabe-Hesketh & Skrondal, 2012). Moreover, candidates are obviously embedded in lists, and lists are embedded in parties. All of this, and the fact that the dependent variable is a proportion, leads us to employ a binomial multilevel regression^4^ with random intercepts for the candidate, list, party, and municipal level.

Our principal explanatory variable of interest is ethnicity. Here we follow political science and economics research on ethnic and racial bias (e.g. Aura & Hess, 2010; Bertrand & Mullainathan, 2004; Fryer & Levitt, 2004; Güell et al., 2014), and create an estimate of candidates’ ethnic identity using their first and last names since ethnicity is not explicitly listed on the ballot materials.^5^ Our approach improves substantially on comparable studies by using a machine-learning algorithm trained on a large dataset using a supercomputer.^6^ We explain it in detail in the online appendix, but wish to highlight five important aspects of our approach here: 1) it is based on a balanced sample of more than 225,000 Croats and Serbs from all Croatian regions; 2) it takes into account people’s first names, last names, as well as their constituting parts in the form of character bigrams; 3) it produces probabilities that a person with a particular first name + last name combination is of Serb or Croat ethnicity^7^; 4) it has a 91.8% success rate of accurately predicting ethnic identity (understood as having a probability of more than 50% of being either a Serb or a Croat) when using names that were not part of the sample training the model (testing set)^8^; and 5) it closely mimics the experience of people from the region when being introduced to a person’s name without knowing their ethnicity, i.e. internally (most often subconsciously) estimating the probability of this person being a Croat or a Serb.^9^ Considering the thoroughness of our approach and the level of accuracy of our model, we believe our analysis has a solid foundation and that our variable *Serb name* is a valid predictor of how voters perceive candidates’ ethnicity.

When it comes to controls on the candidate level, our models include a set of variables shown to have an impact on voters’ decision making: gender, age, incumbency, and distance between the candidate’s place of residence and the municipal center (which we ln-transform to capture the decreasing marginal effect of distance on voting) (Aguilar et al., 2015; Dettman et al., 2017; Devroe & Wauters, 2018; Fulton, 2014; Jankowski, 2016; Matland & Montgomery, 2003; Tavits, 2010). We also include candidates’ positioning on the election lists (*List position*, *First list place*, and *Last list place*), as list placement has been shown to consistently affect voter decision-making (Blom-Hansen et al., 2016; Van Erkel & Thijssen, 2016). We concur with the general view in the literature on preferential voting that list placement can be seen as representing candidates’ ‘quality’ or standing within the party, making this set of variables arguably the most important controls in our analyses. Here it is crucial to note that all of these variables, except incumbency, are explicitly or implicitly in the ballot materials and thus easily visible to voters at polling places. They also account for the vast majority of variables used in studies of voter choice in open list PR systems, which should help strengthen our causal claims.

**Figure 2.**
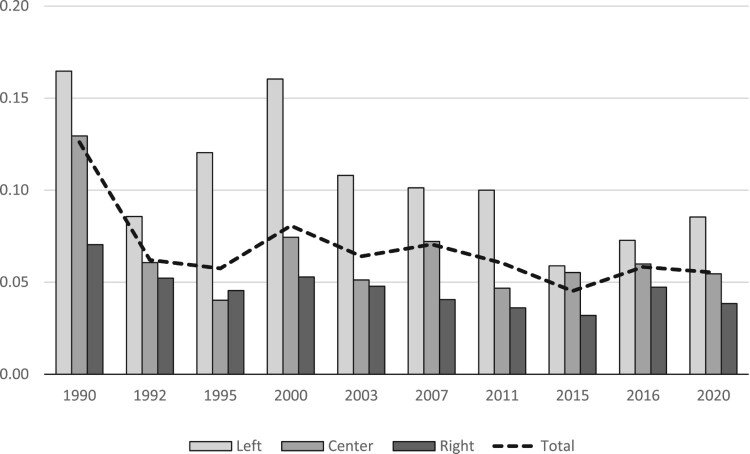
Share of candidates with Serb last names in Croatian elections by party ideology, 1990–2020.

On the level of party lists, we control for the number of candidates with Serb names (i.e. with probabilities of Serb ethnicity being higher than 50%) in order to account for the size of the pool of co-ethnic candidates voters of a particular party in a given district may be able to choose from; total party vote (list + preference votes) in a given municipality; and party list ideology coded after examination of all party programs with 1 being left, 2 center-left, 3, 4 center-right, and 5 right. Figure 2 shows the share of candidates with Serb names on party lists across the ideological spectrum in Croatian elections since the advent of democracy in 1990. What is immediately apparent is that candidates with Serb names are far better represented on the left than on the right. Moreover, their level of overall representation closely follows the census figures. In 1990, for example, there were about 13% of candidates with Serb names, and in the last pre-war census of 1991 12% of Croatian citizens were Serbs. Similarly, in the four elections since 2011, there were about 6% of candidates with Serb names, and in the 2011 census about 5% of Croatian citizens were Serbs. These figures give us additional confidence that our approach is sound.

We also follow the literature on preferential voting when it comes to the municipal level by controlling for variables considered to have possible effects on voters’ propensity to cast preferential votes: average years of education; unemployment; ethnicity; and urbanization^10^ (André et al., 2012; Wauters et al., 2016). Considering our interest in the impact of candidates’ (perceived) Serb ethnicity on voter preferences, we model ethnicity as the proportion of eligible Serb adults (i.e. excluding those who voted in the elections for the special ethnic minority representatives) in the municipal electorate consisting of Croats and Serbs.^11^ We also add a square of this variable to capture its potentially non-linear effects. Additionally, as explained above, we are especially interested in the effects of the local electorate’s exposure to war violence on voter choice. To be specific, we wish to examine whether greater communal exposure to war violence affects the electorate’s propensity to vote for candidates who may be perceived as ethnically Serb. We capture communal exposure to war violence with the rate of disability caused by war operations, which was tallied in Croatia’s 2011 census. Crucially, this variable captures war exposure of the surviving population that currently inhabits the municipalities in question, alleviating modeling problems stemming from migration flows during and after the conflict. In other words, the variable *War disabled* captures exposure to war violence of the average voters in the municipality at the time of the elections in question.^12^ Finally, we account for differences between the 2015, 2016, and 2020 elections by including dummy variables in all our models.

## Determinants of Post-Conflict Ethnic Bias

We present our principal findings in Table 1, with the three models providing tests of our three sets of hypotheses. Our first hypothesis suggested there was a possible bias in the Croatian electorate against ethnic Serb candidates. Here we wish to stress that our research design allows for our propositions to be tested on the intra-party level. In other words, it looks for evidence of ethnic bias in preferential voting *within* party slates, i.e. controlling for voters’ party choice. The results of our Model 1 show we could not find any evidence in support of our first hypothesis. Having a *Serb name* did not lead to any preferential vote penalty for candidates running in Croatian elections. We did find evidence of a slight gender bias, with female candidates getting 0.26 percentage points less than did comparable male candidates. Since the average candidate received 2.2% of preferential votes in a municipality, this is substantively a rather small effect. In line with extensive literature (e.g. Dahlgaard, 2016; Dettman et al., 2017), we also found evidence of modest benefits of incumbency, with former MPs and government ministers getting 1.8 percentage points more than did comparable candidates. What really seemed to matter on the candidate level, however, was the candidates’ geographic closeness to the voters, with the first 100 km of distance shaving 4.4 percentage points off of the candidates’ preferential vote – finding in agreement with the literature on the geography of preferential voting (Gimpel et al., 2008; Jankowski, 2016). As expected (Blom-Hansen et al., 2016; Marcinkiewicz, 2014; Van Erkel & Thijssen, 2016), candidates’ placement on the party lists was also a crucial factor, though obviously we have to note that list placement is not random but a reflection of the candidates’ standing in the party and likely among voters. On the party level, we found preferential voting more used among voters of smaller and right-wing parties; and on the municipal level, we found preferential voting used in more rural communities with higher unemployment and lower levels of education, suggesting that more closely-knit communities under economic and social stress are more likely to use the personal vote. Here we should also note that we undertook a number of robustness checks and found no change in trends among voters of different ideological groups of parties, nor did we find any substantive change in our findings between the three electoral cycles.

**Table 1.** Determinants of preferential vote.

	Model 1			Model 2			Model 3		
	B	S.E.	Sig.	B	S.E.	Sig.	B	S.E.	Sig.
Serb name	−0.013	0.026		−0.054	0.026	*	−0.166	0.026	***
Gender	−0.060	0.011	***	−0.059	0.011	***	−0.059	0.011	***
Age	−0.002	0.002		−0.002	0.002		−0.002	0.002	
Age²	5 × 10^−6^	2 × 10^−5^		4 × 10^−6^	3 × 10^−5^		5 × 10^−6^	3 × 10^−5^	
Distance (ln)	−0.295	2 × 10^−4^	***	−0.295	2 × 10^−4^	***	−0.295	2 × 10^−4^	***
List position	−0.043	0.002	***	−0.043	0.002	***	−0.043	0.002	***
First list place	1.048	0.021	***	1.048	0.021	***	1.049	0.021	***
Last list place	0.458	0.022	***	0.458	0.022	***	0.458	0.022	***
Incumbent	0.303	0.026	***	0.304	0.026	***	0.304	0.026	***
Serb names on list	0.002	0.009		0.002	0.009		0.002	0.009	
Party vote	−0.161	0.004	***	−0.160	0.004	***	−0.159	0.004	***
Ideology	0.035	0.011	**	0.035	0.011	**	0.036	0.011	**
War disabled	−0.052	0.039		−0.055	0.039		−0.274	0.050	***
Serb eligible voters	−1 × 10^−4^	9 × 10^−4^		−0.002	9 × 10^−4^	*	−0.007	0.002	***
Serb eligible voters^2^	3 × 10^−6^	1 × 10^−5^		9 × 10^−6^	1 × 10^−5^		9 × 10^−5^	2 × 10^−5^	***
Unemployment	0.655	0.029	***	0.617	0.029	***	0.604	0.029	***
Education	−0.015	0.007	*	−0.017	0.007	*	−0.018	0.007	*
Urbanization	−0.022	0.004	***	−0.021	0.004	***	−0.021	0.004	***
Election 2016 dummy	0.010	0.023		0.010	0.024		0.010	0.024	
Election 2020 dummy	−1.962	0.034	***	−1.966	0.033	***	−1.966	0.034	***
Serb name x Serb eligible voters				0.014	5 × 10^−4^	***	0.028	0.001	***
Serb name x Serb eligible voters^2^				−6 × 10^−5^	8 × 10^−6^	***	−4 × 10^−4^	2 × 10^−5^	***
War disabled x Serb eligible voters							0.034	0.007	***
War disabled x Serb eligible voters^2^							−7 × 10^−4^	1 × 10^−4^	***
Serb name x War disabled							0.842	0.029	***
Serb name x War disabled x Serb eligible voters							−0.113	0.004	***
Serb name x War disabled x Serb eligible voters^2^							0.002	1 × 10^−4^	***
Intercept	1.479	0.090	***	1.503	0.090	***	1.542	0.090	***
n (Candidates, Parties, Municipalities)	6669 / 97 / 556			6669 / 97 / 556			6669 / 97 / 556		
Δ AIC	−1583633 (−38.28%)			−1585098 (−38.31%)			−1586063 (−38.41%)		

Note: *p < 0.05; **p < 0.01; ***p < 0.001

**Figure 3.**
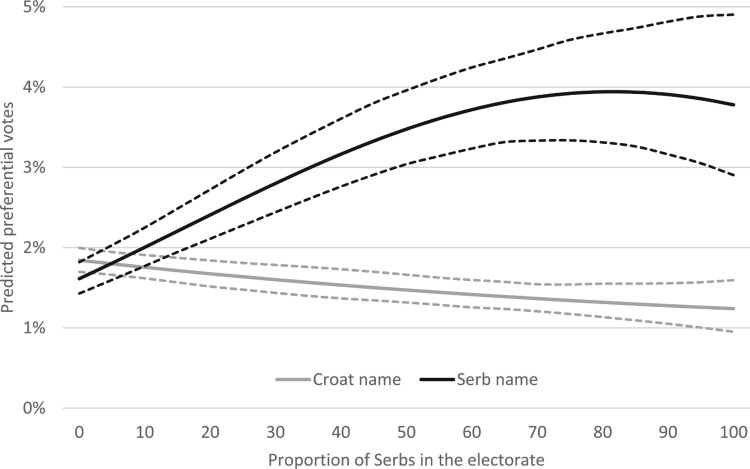
Effect of name ethnicity on preferential votes.

Our second set of hypotheses (H2, H2a, H2b) proposed that any ethnic bias in preferential voting might be conditional on the ethnic makeup of the electorate. The results of our Model 2 provide ample support for these propositions with the coefficients associated with the interactive terms highly statistically significant and in the expected direction. To better understand the nature of this relationship, we show it in graphic form in Figure 3 where we trace predicted preferential votes for candidates with a ‘fully’ Croat name (name suggesting a 100% probability the person is a Croat) and a ‘fully’ Serb name. It closely corresponds to our predictions presented in graphic form in Figure 1. In municipalities with no Serbs in the electorate, candidates with a Serb name could count on 1.61% of the preferential vote, whereas candidates with a Croat name could count on 1.84% – a substantively perhaps small, but noticeable difference. This difference turns to zero at about 5% proportion of Serbs in the electorate – interestingly right around the average proportion of Serbs in the municipal electorate, which stood at 5.6% during this period. In other words, our model suggests there was no ethnic bias in preferential voting in municipalities where the proportion of Serbs in the electorate was close to the national average. As the proportion of Serbs in the electorate increased, however, the pro-Serb bias increased as well – the difference in the preferential vote between our two hypothetical candidates peaking at 2.64 percentage points at the level of Serbs in the electorate of about 85%. Our finding builds on findings from a number of studies in different geographic and temporal contexts that expose ethnic bias among ethnic minorities (e.g. Bergh & Bjørklund, 2003; Boudreau et al., 2019; Landa et al., 1995; Teney et al., 2010). It does that by showing the non-linear nature of this relationship on the communal level that is dependent on the local interethnic balance between the ethnic minority and majority.

**Figure 4.**
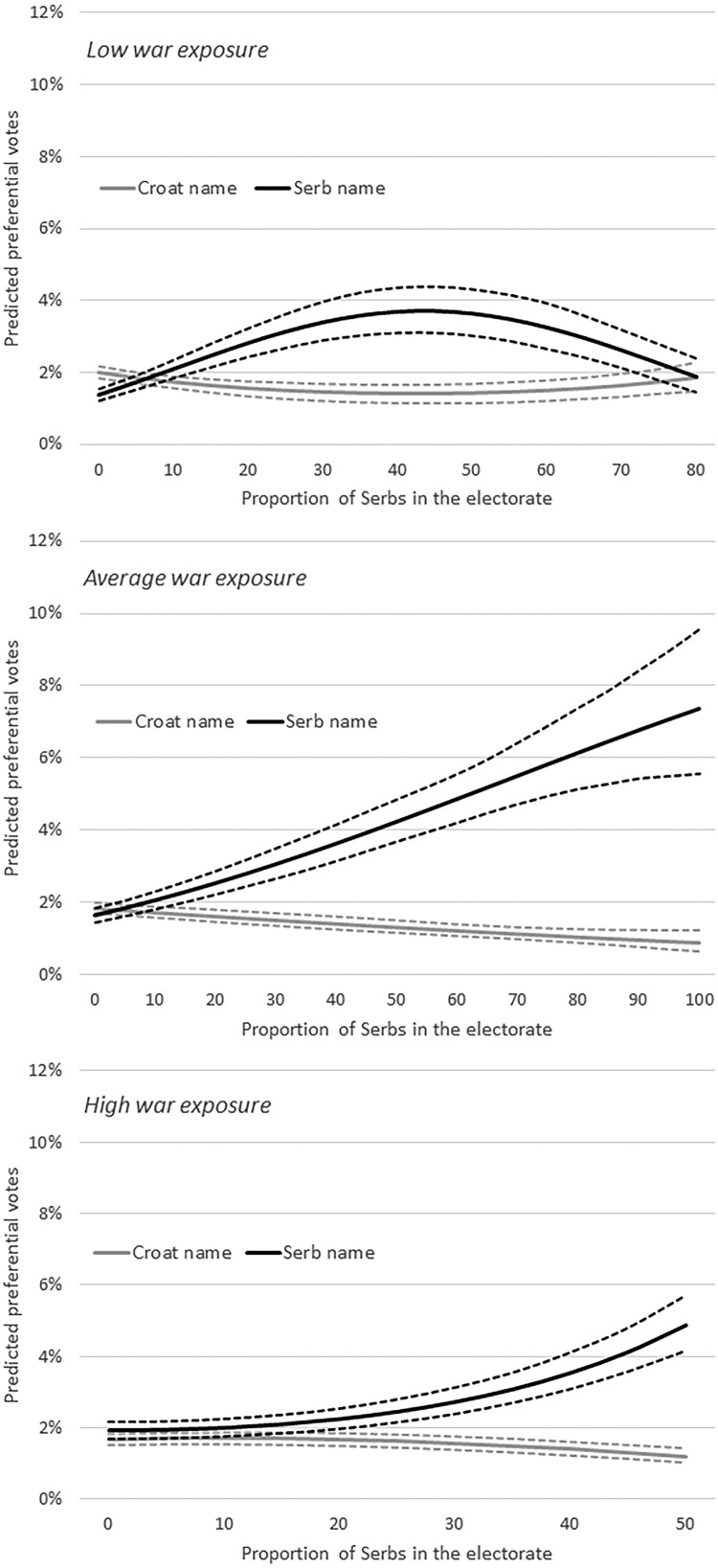
Effect of name ethnicity on preferential votes, conditional on municipalities’ exposure to war violence.

Finally, our third set of hypotheses suggested that this relationship between the electorate’s and the candidates’ ethnicity was contingent on the level of electorate’s exposure to war violence. We expected the level of ethnic bias to be more modest in communities that had little exposure to war violence, and stronger in communities that had a lot of exposure to war violence. This is why our Model 3 interacts the variables capturing candidates’ ethnicity, ethnicity of the electorate, and the exposure of the electorate to war violence. The results of these interactions are all highly significant. Since they do not lend themselves to easy interpretation from the table, we present them graphically in Figure 4. The top panel shows the predicted preferential votes of our two candidates with ethnically clearly defined names in municipalities with low war exposure (one standard deviation below the mean value of the variable *War disabled*), the middle panel shows their predicted preferential votes in municipalities with average war exposure, and the lower panel shows their predicted preferential votes in municipalities with high war exposure (one standard deviation above the mean value of the variable *War disabled*). Readers’ attention should focus not only on the different shapes of the lines predicting the candidates’ preferential votes, but also on the differences in the scales of the x-axes that represent the real-world range of cases in these three groups. In other words, the x-axis in the top graph goes up to 80% because in Croatia there are no municipalities with low war exposure (*War disabled* being at least one standard deviation lower than the mean) that have more than 80% Serbs. Similarly, the x-axis in the bottom graph goes up to 50% because there are no municipalities with high war exposure (*War disabled* being at least one standard deviation higher than the mean) that have more than 50% Serbs. We believe representing the results like this is important to properly understand the nature and the drivers of our findings.

Figure 4 shows some support for our third set of propositions, though with two important caveats. What is clear is that ethnic bias in municipalities with Serb majorities is indeed conditional on the level of their exposure to war violence and rather dramatically so. That is already obvious from the shapes of the three lines showing predicted preferential votes for Serb candidates: parabolic in areas with low war exposure, steeper linear growth in areas with average war exposure, and exponential growth in areas with high war exposure. To be more specific, candidates with Serb names can expect to get a 2.2 percentage point bonus over comparable candidates with Croat names in municipalities with low war exposure and a 50% Serb electorate, a 2.9 percentage point bonus in municipalities with average war exposure and a 50% Serb electorate, and a 3.7 percentage point bonus in municipalities with high war exposure and a 50% Serb electorate. In municipalities with 80% Serb electorate, the differences in these figures would be even more pronounced: no electoral bonus in areas with low war exposure, 5.1 percentage point bonus in areas with average war exposure, and 15.2 percentage points bonus in areas with high war exposure (if municipalities with an 80% Serb electorate and high war exposure existed). All of this is strong evidence in support of our third set of propositions.

The first caveat here is, however, that the same cannot be said of areas with Croat majorities. In fact, our model 3 findings suggest that war-affected areas with Croat majorities showed no ethnic bias. For example, in areas with average and high war exposure with a 100% Croat electorate, preferential votes for Croat and Serb candidates were essentially identical. Some ethnic bias against Serb candidates was present only in areas with overwhelming Croat majorities and *low* war exposure. We believe this is possibly the case because municipalities with overwhelming Croat majorities and low war exposure are predominantly in regions with no history of inter-ethnic contact between Croats and Serbs, whereas the same cannot be said about municipalities with overwhelming Croat majorities and average to high war exposure. We believe it is possible that Croats in these areas of strong Croat majorities and average to high exposure to violence – now safe from possible perceived Serb threat after Croatia’s war victory – exhibit less ethnic bias in spite of recent history of ethnic conflict. More research on the individual level across different Croatian regions, however, would be needed to test this interpretation.

The second caveat to the findings related to our third set of hypotheses concerns the different shapes of the curves predicting preferential votes for Serb candidates, particularly in municipalities with low war exposure. We originally suggested that the curve capturing the decreasing returns to candidates’ ethnic minority identity as the proportion of that ethnic minority in the local electorate increases should be flatter in areas of low war exposure and steeper in areas of high war exposure. That is only partially the case. The two lines showing the predicted preferential votes for candidates with Croat and Serb names are indeed much closer in areas with low war exposure and they get progressively much more divergent as the level of war exposure increases. There are, however, no decreasing returns to Serb ethnicity as the proportion of ethnic Serbs in the local electorate increases in areas with average or high war exposure. This is likely the case because where minority communities experienced war violence, ethnic polarization no longer seems to matter as much – their identity does. We have to be cognizant, however, that the full nature of this relationship cannot be captured by our aggregate-level data. Once again, Figure 4 suggests that our story of ethnicity being the most salient among ethnic minority voters in areas of ethnic polarization may only work in areas unaffected by war violence. Communal experience of war violence may be making ethnicity more salient to members of ethnic minorities everywhere – i.e. *regardless* of local ethnic balance. Much more individual-level empirical research, however, is necessary to properly explain this interaction among interethnic balance, ethnic bias, and experience of violent conflict of ethnic minorities.

Obviously, there is a possibility that our findings’ (conditional) support for the third set of hypotheses could simply be a residual of pre-war trends that do not have anything to do with exposure to war violence. One could reasonably claim that local ethnic violence could have been caused by local ethnic (Serb) bias, rather than the other way around. Knowing the roots of the crisis in Yugoslavia that led to violence, as well as the dynamics of the war in Croatia, we find this possibility of reverse causality unlikely. As many cases in front of the ICTY showed, violence in Croatia was mostly imported and stirred up from the outside (Sambanis & Shayo, 2013). Survey research conducted immediately before the Yugoslav wars suggested relatively low levels of overall ethnic prejudice and bias throughout the federation, with the exception of Kosovo (Sekulić et al., 2006). More importantly, survey research conducted in Croatia immediately before the war suggests there was little connection between ethnic prejudice and the subsequent pattern of violence. We analyzed the results of a survey conducted in late 1989 and early 1990 by a team of local social scientists on a sample of 2158 Croatian citizens (312 of them Croatian Serbs)^13^ and found no connection between the respondents’ level of ethnic prejudice (a composite indicator based on their answers to four related questions about attitudes toward other ethnic groups) and the geographic pattern of violence that followed. To be specific, we split the sample into two groups based on the level of municipal exposure to war violence proxied by the war disability figures. There was no difference in the level of ethnic prejudice between the respondents coming from areas highly exposed to war violence and those coming from areas that avoided exposure to conflict.^14^ Just as was the case with the war in Bosnia and Herzegovina (Costalli & Moro, 2012), the pattern of violence was determined by localities’ strategic value and ethnogeographic location rather than differing levels of local ethnic prejudice or hatred. This gives us confidence that the interpretation of our findings is indeed correct: exposure to war violence intensifies ethnic bias in areas populated by the ethnic minority.

## Conclusions

The majority of civil wars since the end of World War II have been ethnic conflicts (Sambanis & Shayo, 2013). They have been long, deadly, and devastating for the affected communities’ economic and social relations. Although some countries recovering from ethnic conflict manage to return to their developmental paths relatively quickly, virtually all of them suffer from torn interethnic ties, damaged trust, and ethnic discrimination. More often than not, these damaging social dynamics become embedded and entrenched in the political system built after the conflict ends, whether in the form of flawed institutional design, ethnicized political mobilization and competition, or ethnic bias by voters and policy makers. In this article, we used an innovative methodological approach to elucidate the relationship between voter ethnic bias, exposure to ethnic conflict violence, and post-conflict ethnic distribution in a polity where war may have ended more than two decades ago, but the challenges of rebuilding interethnic ties and trust remain.

In addition to creating a novel approach for inferring ethnicity from names, this study contributes to our understanding of voter choice in post-conflict politics. We show that ethnic bias is unequally distributed and is dependent on local interethnic balance. It is more prevalent in communities populated by the ethnic minority. Furthermore, the decreasing rate of return to electoral candidates’ ethnic minority identity as the proportion of ethnic minority voters in the local electorate increases suggests that ethnic bias is likely more prevalent among minority voters in areas of ethnic polarization. Most importantly, we also find the relationship between the ethnic identity of the local electorate and its ethnic bias to be conditional on the level of exposure to war violence. For communities populated by the Serb minority in Croatia, war exposure dramatically increased their electoral ethnic bias. We believe this is likely the case because ethnic violence intensifies the process of withdrawal into a community of co-ethnics among minority voters who have more reason to feel insecure in the post-conflict environment of ethnic tension and discrimination. Finally, the temporal distance between the three electoral cycles our study analyzes and Croatia’s War for Independence shows that the exposure to war violence has a long-lasting effect on voters’ political preferences. This enduring effect gives us pause when we consider the potential for true post-conflict reconciliation in Croatia and other societies recovering from ethnic conflict.

Our study also opens many questions. Most obviously, our aggregate-level analyses need to be supplemented with individual-level studies that will be able to parse out the intricacies of the relationship between voter ethnic bias, experience of violence, and local interethnic balance among members of ethnic minorities and majorities in different settings. Our findings suggest ethnic bias may be highest in areas with highest ethnic polarization (with a higher peak for ethnic minorities than majorities), but this needs to be confirmed with individual-level data. In many ways, the Croatian War for Independence was paradigmatic of the wave of ethnic conflicts happening as the Cold War ended, so we believe the general trends we uncover here have broader relevance and need to be tested in other polities with similar experiences of conflict, but with different types of conflict resolution and overall post-conflict interethnic balance. Also, there is the lingering question of post-conflict institutional design. Proportional representation has been shown to lower electoral ethnicization (Huber, 2012). In that sense, Croatia presents a stringent test case for uncovering voter ethnic bias, in spite of preferential voting provisions in its electoral rules. Further study, however, is needed to uncover how the design of electoral institutions may affect voter ethnic bias in societies recovering from ethnic conflict. Finally, and most importantly, there is the lingering question of the exact mechanism through which ethnic bias becomes perpetuated and embedded in post-conflict societies, making it politically relevant (and often toxic) for decades after the conflict had ended, even among generations that had no direct experience of war violence. In our view, the answers will most likely be found in the interplay between local and national political institutions, as well as parties’ electoral mobilization efforts, with the critical determining factor being the extent to which these institutions and efforts are ethnically defined.

## Supplementary Material

Supplemental Material

## Notes

1 For an excellent review of the literature on the historical legacies – electoral and otherwise – of violence, see Walden & Zhukov, 2021.

2 The only exception was the 2000 election when the HDZ government lowered the number of Serb representatives to one, leading the Serb parties to also (unsuccessfully) run in regular elections.

3 Popularity of preferential voting in countries with open list PR rules has varied from 22% in the 2006 Swedish elections to 64% in the 2010 Latvian elections (André & Depauw, 2017).

4 Some authors (e.g. Allik, 2015; Van Erkel & Thijssen, 2016) instead opt for a linear model with log-transformed vote proportions as the dependent variable. Our robustness checks show both approaches leading to very similar results, which is why we report the findings of the more straightforward binomial model.

5 For an extensive review of the literature relating personal and family names to ethnicity, as well as an innovative approach to deducing ethnicity from names that is different from ours, see Mateos (2014).

6 For a good comparison with a work focused on Croatia, one can consult Jurajda and Kovač (2021). They derive ethnic identity using calendars of the Catholic (Croat) and Orthodox (Serb) churches. Four things are notable about their approach that we improve upon: 1) they only take into account first names; 2) they exclude from analysis all names that do not appear in either of the two calendars; 3) they exclude from analysis all names that appear in both calendars; and 4) they assign first names into binary categories as either Croat or Serb, whereas naming practices in the region are more complex, i.e. many names to different degrees ‘belong’ to both communities.

7 We deliberately exclude from analysis names that clearly belong to other, far less numerous, ethnic minorities: Bosniaks (0.9% in the 2011 census), Italians (0.4%), Albanians (0.4%), and Hungarians (0.3%). Names belonging to these ethnic groups are in an overwhelming majority of cases clearly distinguishable from Croat and Serb names. This simplifies the training of our model and improves its accuracy. We believe that it also better gets at the core question of possible bias against the Serb minority.

8 Here we should note that an overwhelming majority of the names among the 8.2% of our misses were close calls and hovered near the 50% mark. Considering the fact that our variable *Serb name* is a probability score, even these cases should thus not be seen as complete misses.

9 Croats and Serbs share many first and last names, as well as a very similar – some linguists would argue the same, pluricentric – language. Many first and last names, however, dominantly belong to one or the other ethnic group. Our machine-learning algorithm perfectly captures this, with the shared names hovering around 50% and the more monoethnic names having much higher/lower values.

10 We model urbanization as the ln-transformed average weighted settlement size in the municipality, capturing the urban/rural experience of the municipality’s ‘average’ inhabitant.

11 We exclude members of other, far less numerous, minorities like Bosniaks, Italians, Albanians, or Hungarians. This ensures comparability with our analysis of names. As a robustness check, we run models with figures for the other minorities included and find virtually no difference.

12 An alternative measure of exposure to war violence could have been war related deaths. Unfortunately, reliable war death figures disaggregated to the local level in Croatia do not exist. Moreover, death figures would not take into account post-war migration flows, making us confident that *War disabled* is the best possible variable capturing the exposure to war violence of the contemporary municipal population.

13 This survey was used in several studies on the state of pre-war ethnic relations in Croatia (e.g. Kunovich & Hodson, 1999; Sekulić et al., 2006; Strabac & Ringdal, 2008).

14 We are cognizant of the limitations of this approach, but we opted for it due to low numbers of respondents in some municipalities. If we disaggregated the respondents to their 18 municipalities (out of 106 Croatia’s prewar municipalities), the results would have been the same. The correlation between the municipal level of prewar ethnic prejudice and wartime level of violence was only 0.11.
