# Supplementary material for: Ethnic Bias after Ethnic Conflict: Preferential Voting and the Serb Minority in Croatian Elections
Source: Ethnopolitics. 2021 Nov 17;22(1):22–42. doi: 10.1080/17449057.2021.1997440 (PMC9678020; doi:10.1080/17449057.2021.1997440)
Supplement: Supplemental Material [file RENO_A_1997440_SM9240.docx]

**Online appendix: From Names to Prediction of Ethnicity**

We compiled a balanced dataset of people’s full names (i.e. first names and last names) accompanied by their ethnicity and trained a machine-learning (ML) model tackling a classification problem of ethnic affiliation based on features of those names. As ML algorithms require a significant amount of training data, we made use of two large datasets. First, we used the results of the 1964 census of World War II victims from Croatia where census papers that are publicly available included individuals’ names, ethnicity, year of birth, place of birth, as well as circumstances of their death. It is important to note that victims’ data were reported by survivors, most often the victims’ next of kin. Although this may raise questions about data validity, we find it unlikely that data on victims’ ethnicity suffers from systematic problems. The data have been made publicly available online for years (Muzej žrtava genocida, 2021) and we are unaware of any large-scale challenges to the accuracy of their information on ethnicity that would make us question the soundness of our analyses.

Due to the larger number of Serbs than Croats in this resource, we supplemented the 1964 census data by harvesting Croatia’s online phone book, but only in settlements with ethnically homogenous Croat populations (i.e. with 99% or more inhabitants belonging to one ethnic group, so that we could be nearly certain of the ethnicity of the people listed in the phone book). Using the phone book for ethnically homogenous Serb settlements was not an option since inhabitants of these settlements most often do not list their telephone numbers, likely due to fears of harassment. These two sources gave us a perfectly balanced dataset of 225 110 names in total (150 920 from the 1964 census, and 74 190 from the phone book) that we used for training a classification model. What is also really important to note here is that our sample of 225 110 full names covers all regions of Croatia proportionally to the distribution of its Croat and Serb populations. We ensure proportional geographic coverage because last names in Southeast Europe are not only signifiers of ethnicity, but are very much regionally determined. We thus aim to limit any possible regional blind spots that could bias our results. The dataset contains just two columns – full names representing textual data that need to be classified and ethnic affiliation as their assigned labels. After multiple tests using sample subsets and a grid search with various hyper-parameters, we settled on preprocessing rules using character bigrams and a machine-learning algorithm of support vector machine (SVM) with radial kernel.

Although names typically evoke certain ethnicity on the level of words, we believe their constituting parts can be equally informative and can add additional structure to the analyzed textual data. In our view, this captures the common sentiment that a name “sounds” like belonging to a particular community, even though we may have never heard it before. Combination of constituting characters of names as higher n-grams can preserve the ethnic links embedded on the level of a whole name and at the same time capture the infrequent variations that might be otherwise misclassified. We use the full names as well as first and last names separately to create character bigrams as a unique textual input per name associated with specific ethnic class (Serb/Croat).^[[1]](#footnote-1)^ We use these pre-processed names represented as a sparse document-feature matrix as an input for training classification models using support vector machine. The core idea of an SVM algorithm is to find the decision boundary to separate different classes in a dataset with a hyper-plane and maximize their margin (Noble, 2006). Applied radial kernel is a form of transformer helping to generate new features allowing the hyper-plane to be fitted more appropriately (a function that is highly desirable when it comes to unstructured data such as text).

We actually train three models using full names, first names, and last names with a 0.8/0.2 training/testing split. The training itself is done using the R package *caret* (Kuhn, 2019). As the dataset is quite large and requires heavy computational power of a supercomputer, we limit the modelling to five default values of the main parameters [Sigma; Cost] in the *caret* package (parameter tuneLength = 5). For the train control, we use repeated cross-validation with five random splits repeated five times. Apart from produced classes, we also extract class probabilities (treated as a log-odds ratio) using Platt scaling. The performance of each classification model on the validation set is presented in the Appendix Table 1. All models perform well, scoring high above a simple null-model with 50% accuracy based on mere chance of guessing. In our analyses, we obviously opt for the full name model M3 and use it to extract the class probabilities of the names of candidates running in all democratic parliamentary elections held in Croatia since 1990.

**Appendix Table 1: Summary of Classification Models**

|  | Accuracy | F1 | Recall | Precision |
| --- | --- | --- | --- | --- |
| First name (M1) | 0.863 | 0.858 | 0.886 | 0.833 |
| Last name (M2) | 0.885 | 0.883 | 0.900 | 0.867 |
| Full name (M3) | 0.918 | 0.917 | 0.930 | 0.904 |

1. Bigram is a sequence of two adjacent elements from a string of tokens, in this case, characters. For the purpose of this study, we transform raw names to their bigramized versions that are then used as extracted features in machine-learning pipelines. Example: Ivan Horvat -> Iv va an Ho or rv va at. [↑](#footnote-ref-1)
